# Cognitive follow up of a small cohort of Huntington’s disease patients over a 5 year period.

**DOI:** 10.1371/currents.RRN1174

**Published:** 2010-09-07

**Authors:** Sarah L Mason, Ruwani Wijeyekoon, Rachel Swain, Aileen K Ho, Emma L Smith, Barbara Sahakian, Roger A Barker

**Affiliations:** ^*^Cambridge Centre for Brain Repair, University of Cambridge, Cambridge, UK; ^†^Brain Repair Centre, Forvie Site, Robinson Way, Cambridge, CB2 OPY; ^§^University of Reading, School of Psychology, 3 Earley Gate, WhiteKnights Road, Reading, RG6 6AL and ^#^University of Cambridge

## Abstract

A small group of patients with manifest Huntington's disease (HD) were followed longitudinally to assess cognitive decline in relation to time from disease diagnosis. This article looks at performance on a range of computerised and pencil and paper cognitive tasks in patients 5 years post diagnosis, who were assessed annually for a 5 year follow up period. The almost universal cognitive decline reported in other longitudinal studies of HD was not replicated in this study. It was proposed that longitudinal follow up in HD is complicated by the varying degree to which different tasks are able to withstand repeated administration; a finding which would have significant implications on study design in future trials of cognitive enhansing interventions.

## Introduction

Huntington’s disease (HD) is an inherited progressive neurodegerative disorder that is characterised by a triad of motor, cognitive and psychiatric problems [1]. HD pathology is a result of an unstable CAG expansion within the coding region of the Huntingtin gene (Htt), located on the short arm of chromosome 4 [2]. 

Huntington’s disease is thought to affect 6-7 people per 100 000 [3] in the UK. While approximately 30 000 people in the US and 38 000 people in Europe are currently living with HD a further 250 000 people are at risk of having inherited the mutated gene [4].  

HD was traditionally thought to be mainly a movement disorder at least in the early stages and a clinical diagnosis was based solely upon the motor features of the disease. It is now widely accepted that cognitive and psychiatric disturbances are almost universal, begin early in the course of the disease, are heterogeneous and contribute substantially to the functional disability of those affected.  

Anecdotal evidence from families and caregivers along with recent findings suggest that it is the cognitive and psychiatric profile of HD that contributes most to the patient’s quality of life and behavioural functioning, and not their motor phenotype [5] [6]. This is particularly apparent in the pre-symptomatic stages where the first noticeable impairment in functioning is often reported as a loss of ability to continue to work at their highest occupational level. 

Cognitive deficits associated with HD early on in disease are consistent with disruption to the functional integrity of the frontostriatal circuitry [7]. Impairments in executive functioning (the ability to plan, organize and monitor behaviour) are common even in the pre-manifest stages of the disease [8] [9]. The cognitive impairment thereafter is slowly progressive and leads to a profound dementia [10].  

Despite the advances in HD research in the last 20 years, there have been very few major break throughs in terms of medical treatments. The majority of drugs currently used in clinical practice are done so not based on reproducible verifiable evidence but more on anecdotal clinical experience. This is most prominently the case for both the cognitive and psychiatric challenges of HD as, to our knowledge, only one placebo controlled study has been carried out to date that focuses on either of these areas [11]. 

The ultimate hope for new and emerging HD therapeutics is to develop a neuroprotective compound capable of slowing down or even stopping the progression of the disease and ultimately preventing the subtle early signs deteriorating into manifest disease. To date no such drugs have survived clinical testing i.e. none have shown that they can truly slow down disease progression. 

With the absence of any neuroprotective agents there is still a great need for new and more effective symptomatic treatments. While therapeutics designed to treat the cognitive and psychiatric symptoms of HD are undoubtedly the future of HD pharmacological research, we are not yet in a position to achieve this effectively in practice. At present, cognitive performance has only been used as the primary endpoint in one clinical trial where performance was measured using the Mini Mental State Exam (MMSE); a measure of global clinical functioning. In order to see the addition of cognitive and psychiatric endpoints to the protocol of large pharmaceutical studies there is a need to better understand the impairment experienced by patients throughout the course of the disease. Appropriate tools are needed that reliably measure longitudinal change specifically in HD (which may not be the same as those that accurately measure impairment in an HD population cross-sectionally) to stand any hope of demonstrating the efficacy of a cognitive enhancing medication.   

## Methods: 

### Participants:

Eighteen patients were recruited at the Cambridge Centre for Brain Repair, Cambridge UK between 1995 and 2004. They gave informed consent and were enrolled in an ethically approved study of cognition in HD. Of these 5 patients went on to be given fetal neural transplantation as part of a separate study and four patients withdrew their consent without completing 5 years of follow up. These 9 have been excluded from this analysis. 

All patients had mild to moderate HD at time of enrollment as measured by the UHDRS [12] (see Table 1 for a summary of motor characteristics). Patients were excluded if they had a comorbid neurological condition or if they were receiving medication to treat their HD or for any psychiatric condition at baseline. Patients were however permitted to go on treatment during the study if it was deemed clinically necessary. 

### Neuropsychological assessment:

For the purpose of this article patients were grouped according to their time from disease diagnosis. Patients were assessed annually for 5 year period starting at 5 years from disease diagnosis, on the following paper and pencil tasks (in order of administration): 

1) Verbal (letter) fluency: a test measuring the spontaneous production of phonemically related words 

2) Verbal (animal) fluency: a test measuring the spontaneous production of semantically related words.

3) MMSE: A test of global cognitive functioning

4) Stroop Test: a test of executive function that looks at selective attention and interference susceptibility 

5) Symbol Digit Modalities Test: primarily a test of mental speed, motor speed and mental flexibility

6)Retain Trail Making test: a timed test of scanning and visuomotor tracking, divided attention, and cognitive flexibility 

7) Digit span – forwards and backwards: a test of immediate and delayed verbal recall 

8) Rivermead Behavioural Memory Test Battery – the story recall test (immediate and delayed recall): a test of immediate and delayed verbal memory.  

In addition each patient has completed five consecutive years of data on the Cambridge Neuropsychological Test Automated Battery (CANTAB); the battery consisted of the following tasks: 

1) One Touch Stockings of Cambridge – a test of planning and working memory 

2) Spatial Working Memory: tests spatial working memory by measuring the ability to search systematically for hidden tokens among several boxes without returning to boxes where tokens had been found previously 

3) Matching to Sample Visual Search – a test of attention capable of dissociating visual search speed and motor movement speed. 

4) Spatial Span: tests spatial memory span by measuring the ability to memorise the order in which an increasing number of white boxes change colour.****

5) Pattern Recognition Memory: ability to recognize visual patterns following a short delay

6) Spatial Recognition Memory: measures ability to recognize spatial orientation of visual stimuli following a short delay.  

The assessment session took between 1 and 2 hours to complete although this time lengthened as patients became more symptomatic.  

### Assessments:

In addition to the neuropsychological battery all patients were assessed using the motor UHDRS which was completed by a qualified and experienced neurologist.  

### Analysis:

To examine longitudinal decline a mean value of all data points was calculated for each time point. A linear regression model was used with time from diagnosis as the independent variable and patient test performance as the dependent variable.  

## Results:

All longitudinal changes reported are significant to at least the p < 0.05 level. For a detailed summary of test results see Table 1 and Table 2. 

Performance on the verbal fluency task for semantic and phonemic categories declined subtly over the 5 year follow up, as did performance on the interference stage of the Stroop task, the backward span length on the digit span task, total correct on the CANTAB One-Touch Stockings of Cambridge and the total score on the CANTAB Spatial Recognition Memory task.  

A more marked decline, as indicated by the steeper regression slope, was seen in the word reading and colour naming stages of the Stroop task, performance on the Symbol Digit Modalities task and the search time on the CANTAB matching to sample task.  

The remaining tests were not found to deteriorate over time in relation to time from disease diagnosis. 

### Table 1: 

**Table:** 

Test	Score at entry	Slope	P-value
	mean (std dev)	(mean + std dev)	(2-tailed)
Motor assessments			
UHDRS	24.3 (14.4)	1.440 ± 0.3646	0.0168*
TFC	10.7 (2.7)	-0.6532 ± 0.04561	0.0001**
TFA	27.4 (4.0)	0.7726 ± 0.1368	0.0048**
			
General cognition			
MMSE	27.3 (2.1)	-0.1697 ± 0.1751	0.3874
			
Attention & executive function			
Verbal fluency – Letter	31.6 (10.2)	-0.9810 ± 0.2875	0.0270*
Verbal fluency – animals	14.3 (5.6)	-0.5920 ± 0.2026	0.0432*
Stroop colour	52.9 (14.4)	-2.826 ± 0.4607	0.0036**
Stroop word	76 (21.3)	-3.797 ± 0.5605	0.0025**
Stroop interference	32.4 (6.7)	-1.607 ± 0.3493	0.0100**
Symbol digit	33 (11.9)	-2.419 ± 0.2975	0.0012**
Trail Making Test – A	54.8 (29.3)	3.471 ± 3.292	0.3513
Trail Making Test – B	136.6 (75.4)	7.239 ± 5.828	0.2820
Digit Span – Total forward	8 (1.9)	-0.2469 ± 0.09182	0.0547
Digit Span – Total backward	5.3 (1.3)	-0.1697 ± 0.03734	0.0105*
			
Memory			
Rivermead behavioural memory test – immediate	6 (2.1)	-0.2940 ± 0.2509	0.3063
Rivermead behavioural memory test – delay	5.5 (2)	-0.3793 ± 0.2315	0.1767
			
Significance = * p<0.05. ** p<0.01 Abbreviations: UHDRS: Unified Huntington’s Disease Rating Scale, TFC: Total Functional Assessment, TFA: Total Functional Capacity, MMSE: Mini Mental State Exam,			
			
Table 2:			
**CANTAB**	Score at entry	Slope	P-value
	(mean + std dev)	(mean + std dev)	(2-tailed)
Attention & executive function			
OTS – Total score	9.7 (2.7)	-0.5311 ± 0.1169	0.0105*
SWM – Between search errors	39 (26.6)	1.851 ± 0.8235	0.0879
MTS – mean reaction time	1126.9 (363.7)	-35.97 ± 34.90	0.3609
MTS – mean search time	3281.4 (1020.7)	393.8 ± 40.13	0.0006**
SSP – max span	5.1 (1.4)	-0.2116 ± 0.07757	0.0525
			
Visuospatial memory			
PRM – Total correct	18.7 (2.5)	-0.4146 ± 0.1677	0.0688
PRM – Mean latency	2746.4 (636.9)	114.9 ± 127.3	0.4176
SRM – Total correct	15.7 (1.8)	-0.4529 ± 0.09300	0.0082**
SRM – Mean latency	3100.3 (920.5)	29.63 ± 99.42	0.7806
			
Significance = * p<0.05. ** p<0.01 Abbreviations: CANTAB: Cambridge Neurospychological Test Automated Battery, OTS: One-Touch Stockings of Cambridge, SWM: Spatial Working Memory, MTS: Matching to Sample, SSP: Spatial Span, PRM: Pattern Recognition Memory, SRM: Spatial Recognition Memory			

## Discussion:

This study examined the longitudinal cognitive performance in a small cohort of symptomatic HD patients over a 5 year period. As expected, this group of patients showed a significant deterioration in motor performance over this time, as did their cognitive performance, but only in some domains.  

Previous studies have reported an almost universal deterioration in cognitive tests of attention and executive function in longitudinal studies with shorter follow up periods [13] [7].  This study did not replicate these findings; in fact performance was not consistent within domains suggesting that the failure to identify deficits may be a function of poor task sensitivity and specificity during longitudinal follow up. For example, psychomotor speed is often reported as a skill that is susceptible to significant decline early in the course of HD and prone to high rates of decline [7]. Performance on the Symbol Digit Modalities Task and the visual search component of the CANTAB Matching to Sample task support these findings, however, no such decline was identified in the Retain Trail Making Test for either part A or B. Similarly, while performance on the backward digit span deteriorated with advancing disease, this pattern was not replicated by the forward digit span or CANTAB Spatial Span task. 

This lack of consistency could be explained in several ways. Five years is an unusually long time for cognitive follow up and it is likely that prolonged testing, annually, on tasks that remain unchanged, could produce learning effects that may confound the results; i.e. it may be that any deterioration is offset by these learning effects. For example in a task such as the Retain Trail Making Test where the learning is likely to be procedural as apposed to episodic, patients may develop strategies over time that allow them to complete the task more efficiently rather than remembering exact answers. This would allow patient to become artificially good at tasks and render the tasks inept at tapping into any cognitive impairment that may be there. 

Additional, it is highly likely that cognitive performance does not deteriorate in a linear fashion on all tasks. Unfortunately with such a small sample size it is difficult to ascertain whether this is the case in this group of HD patients.        

Getting a clear understanding of cognitive performance over a long period of time in HD is important and potentially vital for future therapeutic trials. While conventional clinical trials tend to only last for 3 years or less, transplantation trials and trials of genetic therapies require longer follow up before being able to conclude the efficacy of such treatments. In addition, clinical studies looking into disease modification in HD will need to be able to demonstrate that the intervention has stopped or slowed the progression of the disease for a significant amount of time. 

Our findings suggest that obtaining a clearer understanding of cognitive change in HD over a long period of time is complex. While some tasks are useful cross-sectionally they may not be helpful longitudinally. Additionally, tasks which are capable of mapping deterioration over a short period of time may not be so reliable for extended follow up periods. It is important to get a good understanding of how cognitive tasks behave over time to allow us to design the best protocols for future disease modifying pharmaceuticals or surgical interventions.  

Funding Information:

This work was initially supported by the MRC.

Competing interests:

The authors have declared that no competing interests exist.
